# Poly(*N*-vinylcaprolactam) and Salicylic Acid Polymeric Prodrug Grafted onto Medical Silicone to Obtain a Novel Thermo- and pH-Responsive Drug Delivery System for Potential Medical Devices

**DOI:** 10.3390/ma14051065

**Published:** 2021-02-25

**Authors:** José M. Cornejo-Bravo, Kenia Palomino, Giovanni Palomino-Vizcaino, Oscar M. Pérez-Landeros, Mario Curiel-Alvarez, Benjamín Valdez-Salas, Emilio Bucio, Héctor Magaña

**Affiliations:** 1 Faculty of Chemical Sciences and Engineering, Autonomous University of Baja California, University Boulevard No. 14418, Otay Mesa, Tijuana 22390, Mexico; jmcornejo@uabc.edu.mx (J.M.C.-B.); kenia.palomino@uabc.edu.mx (K.P.); 2 Faculty of Health Sciences, Autonomous University of Baja California, University Boulevard No. 1000, Tijuana 22260, Mexico; gpalomino@uabc.edu.mx; 3 Institute of Engineering, Autonomous University of Baja California, Benito Juárez Boulevard, Mexicali 21280, Mexico; oscar.manuel.perez.landeros@uabc.edu.mx (O.M.P.-L.); mcuriel@uabc.edu.mx (M.C.-A.); benval@uabc.edu.mx (B.V.-S.); 4 Department of Radiation Chemistry and Radiochemistry, Institute of Nuclear Science, National Autonomous University of Mexico, Mexico City 04510, Mexico; ebucio@nucleares.unam.mx

**Keywords:** medical devices, thermo-/pH sensitivity, graft copolymerization, polymeric prodrug, drug delivery, medical/surgical procedures

## Abstract

New medical devices with anti-inflammatory properties are critical to prevent inflammatory processes and infections in medical/surgical procedures. In this work, we present a novel functionalization of silicone for medical use with a polymeric prodrug and a thermosensitive polymer, by graft polymerization (gamma rays), for the localized release of salicylic acid, an analgesic, and anti-inflammatory drug. Silicone rubber (SR) films were functionalized in two stages using graft polymerization from ionizing radiation (^60^Co). The first stage was grafting poly(*N*-vinylcaprolactam) (PNVCL), a thermo-sensitive polymer, onto SR to obtain SR-*g*-PNVCL. In the second stage, poly(2-methacryloyloxy-benzoic acid) (P2MBA), a polymeric prodrug, was grafted to obtain (SR-*g*-PNVCL)-*g*-P2MBA. The degree of functionalization depended on the concentrations of monomers and the irradiation dose. The films were characterized by attenuated total reflectance Fourier-transform infrared spectroscopy (ATR-FTIR), scanning electron microscopy/energy-dispersive X-ray spectrometry (SEM–EDX), thermogravimetric analysis (TGA), and contact angle. An upper critical solution temperature (UCST) of the films was demonstrated by the swelling degree as a temperature function. (SR-*g*-PNVCL)-*g*-P2MBA films demonstrated hydrolysis-mediated drug release from the polymeric prodrug, pH, and temperature sensitivity. GC–MS confirmed the presence of the drug (salicylic acid), after polymer hydrolysis. The concentration of the drug in the release media was quantified by HPLC. Cytocompatibility and thermo-/pH sensitivity of functionalized medical silicone were demonstrated in cancer and non-cancer cells.

## 1. Introduction

Medical devices are crucial for the success of many medical/surgical procedures today [1]. They are classified based on the type of device, risk, and time of use [2,3]. Based on these characteristics, international health agencies (Food and Drug Administration, FDA) identify them as class I, II, and III devices [4]. Class III devices are high risk, and implantation into patients is long or prolonged [5]. Examples of these devices are coronary stents, pacemakers, and orthopedic and breast implants [6,7,8]. Many of these devices are made with different metallic alloys [9] or polymers [10]. Some of the most widely used polymers are polyvinyl chloride (PVC), polypropylene (PP), polyethylene (PE), and silicone rubber (SR) [11,12,13]. These polymeric materials have been widely used in medical device production due to their thermal and mechanical stability, biocompatibility, cheap production, and compatibility with many sterilization methods [14,15]. Similarly, the production of polymeric medical devices from additive manufacturing (3D printing) has increased due to the development of biocompatible scaffolds, precise device design, and minimal waste of materials during production [16,17,18].

SR is the most exploited material for breast implants due to its exceptional flexibility, extensibility, and biodurability [19], particularly those designed mainly for breast augmentation and reconstruction [20]. Some of the problems reported in patients with breast implants are infections, inflammation, hematomas, and seromas [21,22]. A proposal to solve these problems is to incorporate drugs or biomolecules into the device to achieve a therapeutic effect in the specific site or area. Usually, the medical materials are modified to gain drug-loading capacity [23]. 

Plasma and radiation are methods currently used to modify medical devices [24]. Particularly, the ionizing radiation from Cobalt-60 (^60^Co) serves to modify the surface and mass (bulk) of materials due to the high-frequency energy (gamma rays) to which a material is exposed, producing free radicals for grafting [25,26]. SR has been previously functionalized or copolymerized using direct [27], pre-irradiation [28], and pre-irradiation oxidative methods [29]. The direct method is based on a single step to irradiate the polymer matrix for modification in the presence of one or more monomers [30,31]. This process leads to the formation of free radicals or active sites in the polymer matrix due to high-energy irradiation that favors the initiation of a graft polymerization process [32]. 

Some of the methodological advantages are grafting several polymers in a single step, avoiding conventional catalysts or chemical initiators of a polymerization reaction, and obtaining a sterilized product [33]. Some of these functionalizations have included grafting stimulus-sensitive polymers such as poly(*N*-vinylcaprolactam) [34], poly(4-vinylpyridine) [35], poly(acrylic acid) [36], or poly(glycidyl methacrylate) [37]. 

Some of these polymers have a lower critical solution temperature (LCST), and others have an upper critical solution temperature (UCST) [38,39]. These properties are of great interest in the area of controlled drug release and biomaterials since it allows control in the loading or release of biomolecules under physiological conditions [40,41,42]. These grafted polymers manage to carry and deliver molecules with therapeutic activity, such as diclofenac, ibuprofen, nystatin, and lysozyme, due to noncovalent interactions (van der Waals, ionic, or hydrophobic interactions) [43,44]. 

Another route that has recently been explored is grafting medical devices using polymeric prodrugs, which subsequently can be degraded by hydrolysis, thus releasing the drug [45]. These polymeric prodrugs have advantages over previously described methods since the amount of drug present in the device is a function of the functionalization or graft percentage [46]. Merging more than one approach to functionalize medical devices generates “combined products,” which have seen a significant boom in recent years [47,48]. This method offers numerous advantages, such as a site-specific release and preventive or prophylactic effects, and minimizes the first-pass effect of drugs [49,50,51]. The design and research of new “combined products” must focus on the devices being stable for biological applications and capable of responding to physiological stimuli such as pH and temperature. 

In this context, the goal of this work was the modification of medical-grade silicone firstly with PNVCL, a temperature-sensitive polymer, and secondly with the polymeric prodrug P2MBA. The synthesized copolymer, (SR-*g*-PNVCL)-*g*-P2MBA, was characterized by ATR-FTIR, TGA, SEM–EDX, contact angle, swelling, and responsiveness to temperature. The degree of salicylic acid release by hydrolysis was evaluated at different pH values and temperatures by HPLC (quantitative–qualitative determination) and GC–MS (qualitative determination). Finally, the cytotoxicity of (SR-*g*-PNVCL)-*g*-P2MBA was evaluated in cancer and non-cancer cells.

## 2. Materials and Methods

### 2.1. Materials

SR of a 1 mm thickness was obtained from Goodfellow Cambridge Ltd (Huntingdon, UK). It was cut into 5 × 2 cm^2^ films. The SR films were washed with ethyl alcohol to remove impurities for 2 h. Subsequently, the films were dried under a vacuum at a temperature of 60 °C overnight. *N*-vinylcaprolactam 98% was obtained from Sigma-Aldrich (St. Louis, MO, USA) and purified by vacuum distillation. Salicylic acid, dimethylaminopyridine (DMAP), methacrylic anhydride, triethylamine, ethyl ether, dichloromethane (CH_2_Cl_2_), p-dioxane, ethanol, magnesium sulfate, and petroleum ether (2MBA synthesis reagents) were acquired from Sigma-Aldrich. The reagents for HPLC (acetonitrile and methanol) were purchased from Sigma-Aldrich. A Hypersil GOLD C18 column (250 × 4.6 mm^2^) was obtained from Thermo Scientific (Waltham, MA, USA). A 3-(4,5-dimethylthiazol-2-yl)-2,5-diphenyltetrazolium bromide (MMT) kit was obtained from Roche (Basel, Switzerland). 

#### Synthesis of 2MBA

The synthesis was achieved by placing DMAP (0.49 g) (4 mmol), salicylic acid (6.91 g) (50 mmol), Et_3_N (8.42 mL), and CH_2_Cl_2_ (20 mL) in a round-bottomed flask (100 mL). Methacrylic anhydride (7.50 g) (50 mmol) was added little by little below 0 °C and with constant stirring conditions. The organic phase of the reaction was extracted with ethyl ether and recrystallized in vacuo. The precipitate (monomer or 2MBA) was purified in petroleum ether and p-dioxane and characterized by ^1^HNMR and ATR-FTIR [52].

### 2.2. Grafting Process 

#### 2.2.1. Synthesis of SR-g-PNVCL (First Functionalization) 

SR films (1 × 2.5 cm^2^) were functionalized with *N*-vinylcaprolactam by a direct graft method [53]. This process was carried out by irradiating the silicone films using a cobalt (^60^Co) source, Gammabeam 651 PT, MDS Nordion, Ottawa, ON, Canada. Briefly, the films were placed in glass ampoules in a solution of monomer (NVCL) in toluene. The ampules were degassed, closed, and irradiated to initiate a graft polymerization process. The films were removed from the ampules and washed with ethanol and water to remove monomer or homopolymer impurities from the reaction. SR-*g*-PNVCL films were dried under a vacuum, and the percentage of grafting was calculated.

#### 2.2.2. Synthesis of (SR-g-PNVCL)-g-P2MBA (Second Functionalization)

Similarly, the SR-*g*-PNVCL films were placed in glass ampoules in a concentrated monomer solution (1 M/toluene) and exposed to conditions of a direct method using irradiation doses of 5, 10, 20, 30, 40, and 50 kGy. These experiments were essential to determine if the graft grade was a function of dose. Furthermore, the monomer concentration effect was evaluated, using concentrations of 0.0125, 0.025, 0.05, 0.10, 0.25, 0.50, and 0.75 M (in these experiments, a dose of 5 kGy was established). For both experiments, at the end of the procedure, the ampules were opened, and the films were removed, washed with ethanol, and dried under vacuum. For the first and second grafting processes, the graft was calculated using Equation (1) [43]:

(1) $$\mathrm{Grafting}\;\left(\%\right)=\;\frac{W_{F}-W_{I}}{W_{I}}\times 100$$

where $w_{F}$ is the weight of the grafted film and $w_{I}$ is the initial weight.

### 2.3. Characterization of (SR-g-PNVCL)-g-(P2MBA) Films

#### 2.3.1. ATR-FTIR Analysis

The SR, SR-*g*-PNVCL, and (SR-*g*-PNVCL)-*g*-P2MBA films were characterized using ATR-FTIR, at a range of 4000–650 cm^−1^ with 16 scans using a Perkin–Elmer Spectrum 100 spectrometer (Perkin Elmer Cetus Instrument, Norwalk, CT, USA).

#### 2.3.2. TGA Analysis

The modified and unmodified films (15–20 mg) were characterized by thermogravimetric analysis using TGA Q50 equipment (TA Instruments, New Castle, DE, USA). These experiments were achieved under inert atmosphere. The heat rate was 10 °C/min from 25 to 800 °C. 

#### 2.3.3. SEM-EDX Analysis

The SR, SR-*g*-PNVCL, and (SR-*g*-PNVCL)-*g*-P2MBA films of 0.5 × 1 cm^2^ were analyzed by SEM-EDX. The film surfaces and cross-sections were observed to identify possible functionalization sites due to the graft polymerizations. A JSM-6010LA (JEOL, Tokyo, Japan) instrument was used under low-vacuum conditions (30 Pa) and an acceleration voltage of 15 kV.

#### 2.3.4. Contact Angle, Swelling, and Temperature-Responsive Analysis

The unmodified and modified films’ contact angle was evaluated by placing water droplets on different spots of the surface area. A Kruss DSA 100 device (Matthews, NC, USA) was used. Limit swelling tests were performed by placing films in 20 mL of water at room temperature. The films were weighed before placing them in water and subsequently weighed at 10, 20, 40, 50, and 60 min. The swelling percentage was determined as follows [43]:

(2) $$\mathrm{Swelling}\left(\%\right)=\frac{W_{2\;}-{\;W}_{1}}{W_{1}}\times 100$$

where $w_{2}$ and $w_{1}$ represent the weights of the swollen film and dried film, respectively.

The thermal sensitivity of the (SR-*g*-PNVCL)-*g*-P2MBA films was determined by weighing dry films and subsequently swelling them in 20 mL of distilled water for 24 h at different temperatures (28–34 °C). The weight difference was found based on Equation (2). 

### 2.4. Drug Release and Characterization Analysis (HPLC and GC–MS)

SR-*g*-PNVCL and (SR-*g*-PNVCL)-*g*-P2MBA) films (7.6% and 33.5%) (0.5 × 1 cm^2^; 20–25 mg) were placed in phosphate-buffered saline (PBS) at pH 5.5 and 7.4 (5 mL) at 50 RPM and 37 °C (physiological pH levels and temperature) in a Mini Shaker VWR (West Chester, PA, USA). Aliquots were taken at different sampling times, and subsequently, the samples were quantified by HPLC using Ultimate 3000 Thermo scientific equipment (Waltham, MA, USA). The separation mechanism was by reverse-phase RP-HPLC, using a Hypersil COLD Column (Thermo Scientific) (250 × 4.6 mm^2^), octadecyl silica type (ODS or C18). A sample injection volume of 10 µL was used, with a column temperature of 40 °C, using a mobile phase of PBS and acetonitrile (70:30), with a flow rate of 1 mL/min. Finally, a photodiode array detector was used. The concentration was determined by performing calibration lines of salicylic acid of a known concentration at a wavelength of 235 nm. The amount of salicylic acid present or grafted on the film (SR-*g*-PNVCL)-*g*-P2MBA was determined by Equation (3) [45]:

(3) $$\mathrm{Salicylic}\;\mathrm{acid}\;\left(\mathrm{mg}/g\right)=\frac{\mathrm{Grafting}\;\left(\%\right)}{\left(100+\mathrm{Grafting}\left(\%\right)\right)\;x\;\left({\mathrm{MW}}_{2\mathrm{MBA}}\right)\;}\times \left({\mathrm{MW}}_{\mathrm{Salicylic}\;\mathrm{acid}}\right)\times 1000$$

The hydrolysis–release process was evaluated by the first- and zero-order models. Statgraphics Centurion 18 software (version 18.1.13 2020, Statgraphics Technologies Inc., The Plains, VA, USA) [54] was used for this modeling. The salicylic acid HPLC retention time of the problem samples was compared with a standard. The salicylic acid molecular ion was also identified from the release media employing gas chromatography–mass spectrometry (GC–MS). The release medium was exposed to ethyl ether to carry out extraction of the organic phase. The ethyl ether was evaporated, and the precipitate was resuspended in methanol. This solution was injected into a Thermo Scientific TRACE 1310 gas chromatograph model. This gas chromatograph was coupled to a mass spectrograph (single quadrupole), namely, the Thermo Scientific ISQ LT model. Helium was used as a mobile phase at a flow rate of 1 mL/min. A temperature ramp of 40 °C/min was carried out until reaching a temperature of 280 °C.

### 2.5. Cytocompatibility Analysis

Murine embryonic fibroblast cell line BALB/3T3 (ATCC CCL-163, Manassas, VA, USA) and human cervical cancer cell line HeLa (ATCC CCL-2, Manassas, VA, USA) were used to determine the viability percentage after exposure to the modified materials as we previously reported. Briefly, the assays were carried out in 96-well plates seeded with 3000 cells per well in Dulbecco’s modified Eagle’s medium (DMEM) medium with FBS (Fetal bovine serum, 10% *v*/*v*), penicillin–streptomycin (1% *w*/*v*), and gentamicin (10 μg/mL) for 12 h in culture standard conditions. The SR, SR-*g*-PNVCL, (SR-*g*-PNVCL)-*g*-P2MBA 1% (graft percent), (SR-*g*-PNVCL)-*g*-P2MBA 7%, and SR-*g*-PNVCL)-*g*-P2MBA 17% (0.25 × 0.20 cm^2^) films were submersed in the cell media and incubated in a humidified atmosphere of 5% CO_2_ at 37 °C for 24 or 48 h. On the contrary, for the functional characterization after 12 h of incubation, the SR, SR-*g*-PNVCL, SR-*g*-(PNVCL/P2MBA) 1%, SR-*g*-(PNVCL/P2MBA 7%, and SR-*g*-(PNVCL/P2MBA 17% (0.25 × 0.2 cm^2^) films were placed in direct contact with the cell media and maintained in standard conditions of 5% CO_2_ at 37 °C and adequate humidity for 12 h. After this time, the incubator temperature was set to 30 °C for a soft temperature change overnight, and the cell viability was analyzed at 48 h. The films were removed, and an MTT kit was used to quantify the cell viability. Cells without films were used as a negative, and all experiments were performed in triplicate. Lastly, the absorbances were measured by spectrophotometry at 620 nm (Multiskan FC, Thermo Scientific). The statistical data evaluation was done by one-way ANOVA using GraphPad Prism 7 (San Diego, CA, USA). The cytocompatibility was determined as follows Equation (4) [46]:

(4) $$\mathrm{Cytocompatibility}\;\left(\%\right)=\;\frac{{\mathrm{Abs}}_{\mathrm{Sample}}}{{\mathrm{Abs}}_{\mathrm{Control}}}\cdot \;100$$

## 3. Results

### 3.1. Grafting Process

#### 3.1.1. Synthesis of SR-g-PNVCL 

**Figure 1:**
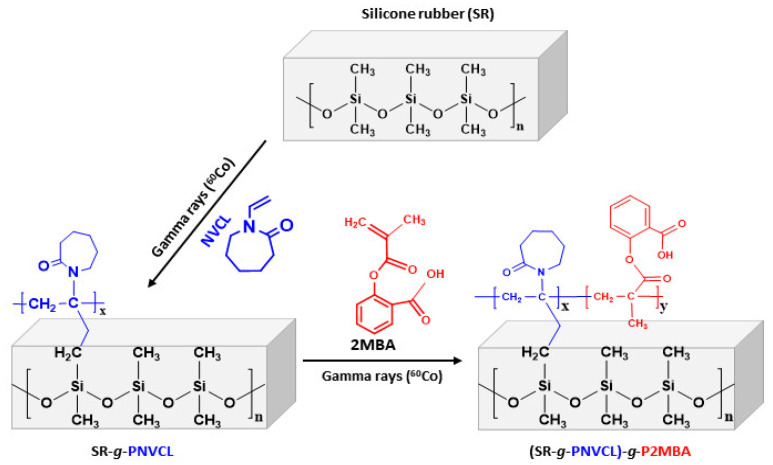
Functionalization of silicone rubber (SR) with NVCL and 2MBA by gamma radiation method.

The functionalization of PNVCL in the silicone films (Figure 1) was carried out successfully according to the previously reported method [53]. The films were irradiated at 50 kGy and in 50% monomer (NVCL) toluene solutions. 

Toluene played a critical role in this phase, as it helped the swelling of the films, thus favoring the migration of the monomeric solution throughout the silicone polymeric matrix [55,56]. The obtained results were consistent with the literature, in which a graft percentage between 37 and 38% was obtained [53]. In this way, the first phase of modifying the silicone polymer matrix (SR-*g*-PNVCL) was performed.

#### 3.1.2. Synthesis of (SR-g-PNVCL)-g-P2MBA

**Figure 2:**
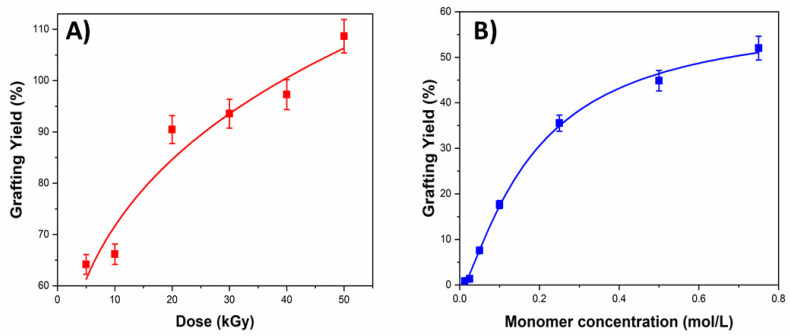
Grafting percentages of 2MBA onto SR-*g*-PNVCL films as a function of (**A**) monomer concentration and (**B**) absorbed dose.

Once the SR-*g*-PNVCL films were synthesized, experiments were carried out to as-sess the factors that could influence the degree of grafting of the proposed new monomer (2MBA) (Figure 1). A monomer concentration (1 M) was established, and the irradiation dose was varied between 5 and 50 kGy (Figure 2A). An increase in the graft grade (64 to 108.6%) was indeed found with the proposed doses because the higher the energy of radi-ation to which the polymeric matrix (SR-*g*-PNVCL) is subjected, the more radical sites there are to promote a graft polymerization process [57]. Once it was shown that the dose effect was essential to moderate the degree of grafting, the concentration of the monomer in the reactions was varied. An amount of 5 kGy and monomer concentrations between 0.0125 and 0.75 M were established. Graft percentages between 0.816 and 52.01% were observed (Figure 2B), thus demonstrating that the higher the monomer’s concentration in the solution, the greater the graft capacity [56]. With the method above, it was possible to establish the monomeric concentration essential to control the films’ degree of functionalization.

### 3.2. Characterization of (SR-g-PNVCL)-g-P2MBA Films

#### 3.2.1. ATR-FTIR Analysis

**Figure 3:**
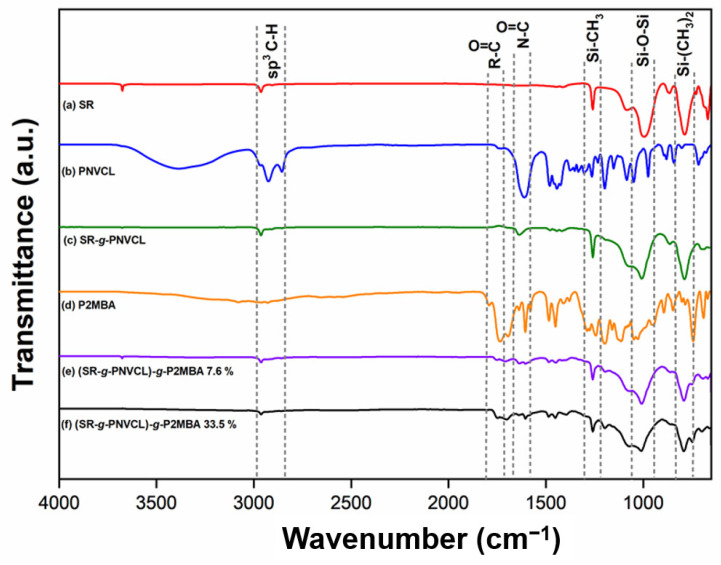
ATR-FTIR spectra of SR (**a**), PNVCL (**b**), SR-*g*-PNVCL (**c**), P2MBA (**d**), (SR-*g*-PNVCL)-*g*-P2MBA 7.6% (**e**), and (SR-*g*-PNVCL)-*g*-P2MBA 33.5% (**f**).

Figure 3 shows the spectra’s chronology until obtaining the target copolymerized system (SR-*g*-PNVCL)-*g*-P2MBA. First (Figure 3a), with SR as the base matrix for the synthesis, characteristic bonds were observed, such as at 2964 cm^−1^, corresponding to the C-H sp^3^ stretching mode; at 1257 cm^−1^, a band corresponding to the Si–CH_3_ stretch; at 997 cm^−1^, the stretch of Si–O–Si; at 820 cm^−1^, a signal corresponding to the Si–(CH_3_)_2_ bending mode [58]. The characterization of the PNVCL (homopolymer), was carried out, this being the polymer to be grafted onto the silicone. PNVCL presented characteristic bands (Figure 3b), such as at 2923 cm^−1^, corresponding to the C–H sp^3^ stretching mode, at 1625 cm^−1^, an intense band corresponding to amide carbonyl (N–C=O), and at 1479 cm^−1^, a band corresponding to the C–N stretch [59]. In Figure 3c, the evidence of the first functionalization (SR-*g*-PNVCL) can be observed. At 1635 cm^−1^, the stretching of the amide carbonyl (N–C=O), corresponding to PNVCL, as well as the C–N stretch band at 1477 cm^−1^, can be observed. Bands at 1265, 998, and 821 cm^−1^, corresponding to stretching of Si–CH_3_, Si–O–Si, and Si–(CH_3_)_2_, can be observed, all the previous signals proper to silicone, ensuring the first functionalization [53]. In Figure 3d, bands of the P2MBA (homopolymer), the second polymer proposed for functionalization, can be observed. At 2945 cm^−1^, a signal corresponding to C–H sp^3^ is present, and between 3100 and 2800 cm^−1^, there is a broad band attributed to the O–H stretching of the carboxylic acid. At 1724 and 1710 cm^−1^, bands corresponding to the C=O stretch of the ester groups, carbonyls and carbolic acid, can be observed. At 1600 cm^−1^, bands correspond to the C=C stretch of the aromatic region of P2MBA, and at 960 cm^−1^, there is an intense band corresponding to the C–H bending (out of plane) aromatic di-substitution in the *ortho*-position [52]. 

Finally, in Figure 3e,f, bands of Si–CH_3_, Si–O–Si, and Si–(CH_3_)_2_ at 1265, 998, and 821 cm^−1^ can be seen, corresponding to SR. At 1725 cm^−1^, carbonyl bands (C=O) can be observed, attributed to the presence of P2MBA, thus demonstrating that the second proposed functionalization of (SR-*g*-PNVCL)-*g*-P2MBA was achieved.

#### 3.2.2. TGA Analysis

**Figure 4:**
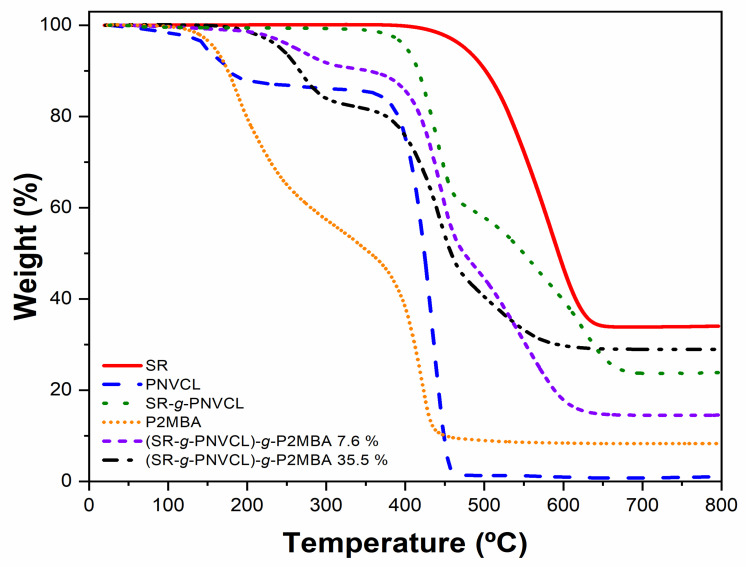
TGA thermograms of SR, PNVCL, SR-*g*-PNVCL, P2MBA, (SR-*g*-PNVCL)-*g*-P2MBA 7.6%, and (SR-*g*-PNVCL)-*g*-P2MBA 33.5%.

Thermogravimetric analyses (Figure 4) were carried out to appreciate the different end stabilities of the polymers involved and the changes that may arise in the proposed synthesized system of (SR-*g*-PNVCL)-*g*-P2MBA. In the first phase of copolymerization, SR and PNVCL were involved. Figure 4 shows how SR presents a single thermal degradation at 517 °C [53]. On the contrary, PNVCL presents a thermal degradation at 434 °C [59]. Consequently, in the first functionalization carried out (SR-*g*-PNVCL), two degradations can be observed, the first between 385 and 452 °C (corresponding to PNVCL) and the second from 452 to 600 °C (corresponding to SR), demonstrating a correct functionalization of SR-*g*-PNVCL, as well as a correlation with previously described infrared experiments that demonstrate the graft [44]. The second stage of functionalization was carried out with P2MBA, as shown in Figure 4. Two thermal decompositions can be observed, the first at 221 °C and the second at 393 °C [45]. In the (SR-*g*-PNVCL)-*g*-P2MBA samples with a graft grade of 7.6 and 33.5%, different intervals of thermal decompositions can be observed. In the sample with a graft of 7.6%, a first decomposition phase occurred between 220 and 350 °C, attributed to P2MBA and PNVCL. Compared to the film with the highest graft (33.5%), this first phase has a decrease between 220 and 320 °C; this is because the higher the graft percentage, the more significant the similarity to the homopolymer.

The second degradation phase for the graft of 7.6% occurred between 400 and 450 °C, and the third between 450 and 600 °C, attributed to SR. The results of these experiments demonstrate that grafting was achieved in two stages, as we were able to observe previously in infrared tests and as, depending on the percentage of P2MBA graft, we could increase or decrease the stability of the proposed system. These results indicate the potential of this final system (SR-*g*-PNVCL)-*g*-P2MBA, with grafts in the order of 7.6 to 33.5% for biomedical applications, as they would not suffer any degradation or modification since the temperature body temperature is around 37 °C [60].

#### 3.2.3. SEM-EDX Analysis

**Figure 5:**
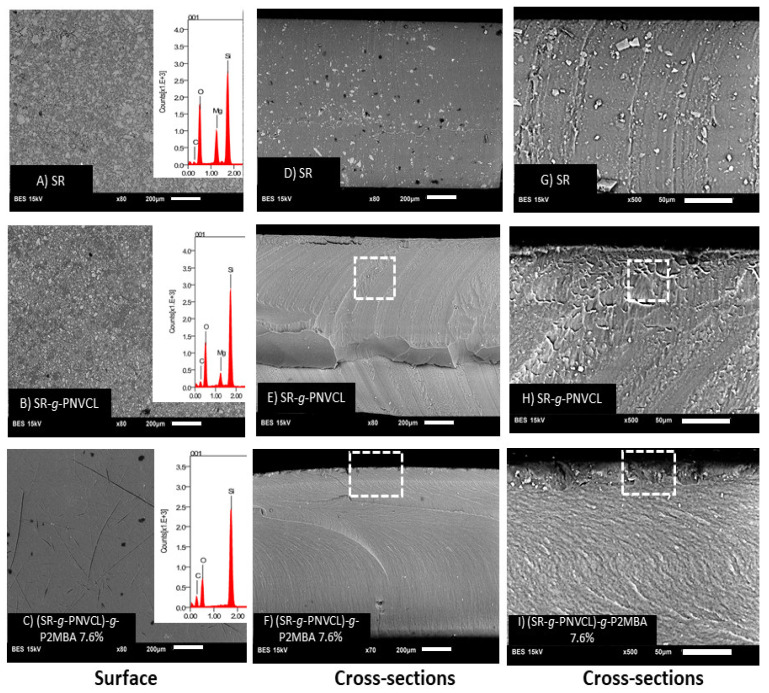
SEM images of the surface of (**A**) SR, (**B**) SR-*g*-PNVCL, (**C**) (SR-*g*-PNVCL)-*g*-P2MBA 7.6%, and the cross-section of SR, SR-*g*-PNVCL, and (SR-*g*-PNVCL)-*g*-P2MBA 7.6% (**D**–**F**) (200 µm); (**G**–**I**) (50 µm)). Dashed line square indicates grafting areas.

After spectrophotometric (Figure 3) and thermogravimetric (Figure 4) characterizations that demonstrated the grafting of the final synthesized system, characterization was carried out by scanning electron microscopy (Figure 5) in order to be able to identify changes in the topography of the films and to determine if the graft of PNVCL and P2MBA was made on the surface of the films or in bulk. Figure 5A shows the surface of SR, in which irregular and traditional topography can be observed, as previously reported [28,61]. EDX analysis showed carbon, oxygen, silicon, and magnesium atoms [62]. Magnesium and aluminum metals were previously reported in the literature and play a vital role in synthesizing silicone for the vulcanization process [63,64]. The surface of SR-*g*-PNVCL (Figure 5B) shows morphology similar to that of SR, but through EDX, there was an increase in the percentage of carbon mass (10%) due to the PNVCL graft. In Figure 5C, the film’s surface can be observed, referring to the second grafting stage to obtain (SR-*g*-PNVCL)-*g*-P2MBA. The second functionalization showed a change in the film’s topography, with a smooth surface compared with the previous ones. This effect was previously observed, and it demonstrates that the P2MBA graft was on the film’s surface [46]. The EDX analysis (Figure 5A–C) showed an increase of 24% in the mass percentage of carbon regarding SR and of an additional 14% for SR-*g*-PNVCL, attributed to the grafted P2MBA.

Later, the morphology of the films was observed in the cut section. In Figure 5D, which corresponds to SR, an irregular morphology with traditional dark deposits can be observed [65]. When the PNVCL graft was performed, a total change in the transversal section’s morphology occurred, as seen in the Figure 5E. These changes have been previously reported and demonstrate how the PNVCL graft was carried out in the same way in bulk [66]. Carrying out the graft polymerization process in toluene produces swelling of the silicone films and favors bulk grafting [55]. In Figure 5F, a regular morphology can be observed compared with the previous ones, as well as the formation of a new layer in the upper part. The layer can be appreciated with a more significant enlargement of the cross-sectional area (Figure 5I). This effect is characteristic and was previously observed in film grafts [45]. It is important to differentiate this from Figure 5H, referring to SR-*g*-PNVCL, where the layer can also be observed in the upper part but is less than in (SR-*g*-PNVCL)-*g*-P2MBA. SR-*g*-PNVCL cross-section films can be seen, characteristic of the fact that the graft was also being carried out in bulk. In the silicone cross-section (Figure 5G), the graft’s layer and different morphologies from the two functionalizations cannot be observed.

#### 3.2.4. Contact Angle, Swelling, and Temperature Responsiveness Analysis

**Figure 6:**
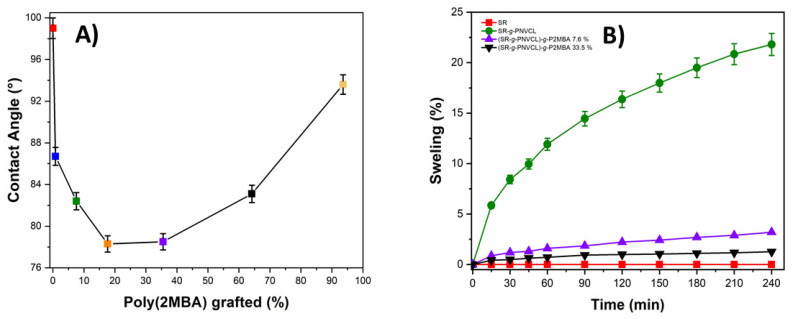
(**A**) Water contact and (**B**) swelling profile of SR and (SR-*g*-PNVCL)-*g*-P2MBA films.

**Figure 7:**
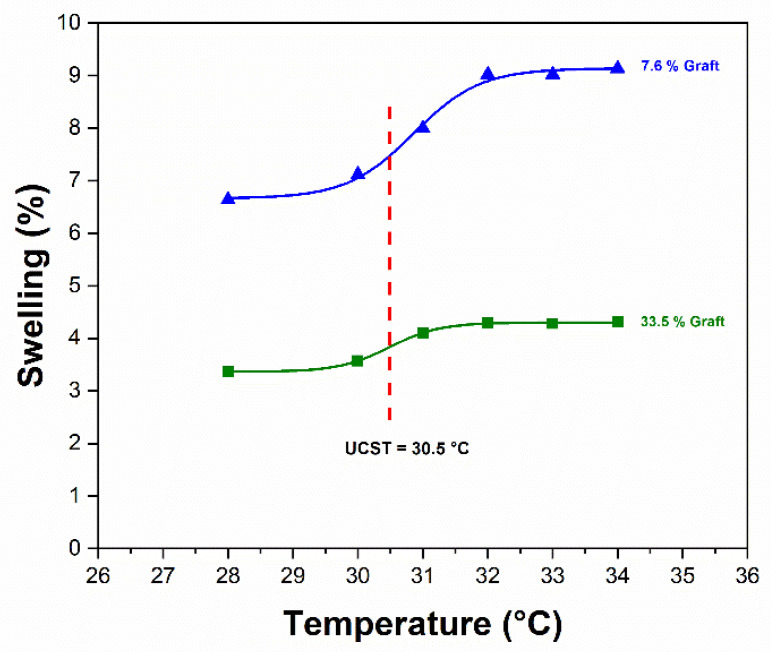
Temperature response by swelling of (SR-*g*-PNVCL)-*g*-P2MBA films.

Contact angle analysis was carried out to determine the hydrophobic or hydrophilic nature of the material. Thus, the behavior of the films could be predicted when in contact with physiological aqueous media. Figure 6A shows how SR presented a contact angle of 99°, demonstrating a hydrophobic material attributed to methyl groups distributed throughout the polymer chain [67,68]. Once the first functionalization (SR-*g*-PNVCL) was carried out, the films presented a contact angle of 90°. This decrease is due to the polar groups of PNVCL, which favor hydrophilic interaction with aqueous media [44]. The P2MBA graft in the order of 0.816–33.5% showed a decrease in the contact angle of 86.7°–78.8°. This decrease in contact angle is attributed to the polar groups (carboxylic acids) present in P2MBA [69]. At high graft percentages (64.1% and 93.8%), an increase in the contact angle (83.1° and 93.6°) was observed; this is attributed to the fact that the greater the graft, the greater the intramolecular interactions of P2MBA, causing a hydrophobic effect [70]. A swelling test was also performed (Figure 6B). The swelling of the films was observed over time, with silicone, due to its previously described hydrophobic characteristics, exhibiting zero swelling up to 240 min. Films with the first copolymerization (SR-*g*-PNVCL) showed a constant increase in the percentage of swelling over time, with a swelling of up to 21%. This swelling was due to an increase in the film’s hydrophilicity, attributed to the grafted polar groups of PNVCL. The (SR-*g*-PNVCL)-*g*-P2MBA films, with 7.6 and 33.5% grafts, showed swelling of 3.20 and 1.26%, respectively, demonstrating that the higher the P2MBA graft, the lower the swelling. These results favorably indicate that the material, when used in biomedical applications, would not undergo swelling processes at high grafting percentages, which could result in deformation or damage to other materials in a medical device [46]. The swelling degree as a function of temperature was also investigated. This experiment aimed to detect the swollen/unswollen transition temperature of the films (Figure 7).

PNVCL is a well-known thermoresponsive material with an LCST close to the body temperature [71]. Interestingly, PNVCL grafted on SR presents a UCST instead of an LCST [45]. The transition was maintained for copolymeric grafts of PNVCL and ionizable monomers [32,72]. It is noteworthy that only a few polymers present a UCST, which is attributed to the weakening of the associative interactions among polymer chains when the temperature is above a certain threshold, which favors the contact of individualized chains with the aqueous medium [73,74]. In our work, the (SR-*g*-PNVCL)-*g*-P2MBA films with 7.6 and 33.5% grafts showed a UCST at 30.5 °C (Figure 7). This phenomenon is relevant in the hydrolysis of the drug, as discussed below.

### 3.3. Drug Release and Characterization Analysis (HPLC and GC–MS)

**Figure 8:**
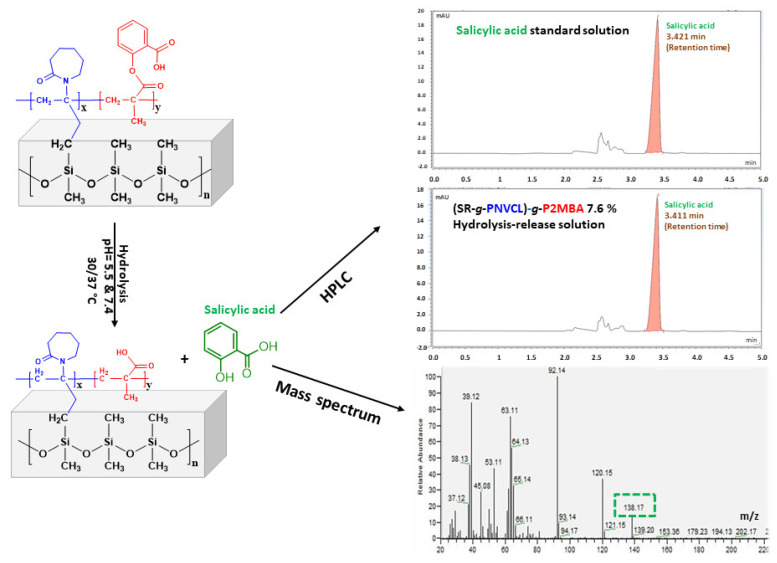
Hydrolysis–release of salicylic acid from (SR-*g*-PNVCL)-*g*-P2MBA films, HPLC quantification, and molecular ion detection.

In the HPLC chromatogram in Figure 8, the retention time at 3.421 min corresponds to the peak of the salicylic acid (standard solution) and at 3.411 min, the peak of the salicylic acid release samples occurred at pH = 7.4, which confirms that salicylic acid is present in the investigated sample. Additionally, a release sample (previously extracted with organic solvent) was injected into the GC–MS. The MS spectrum presents the molecular ion of salicylic acid (138.17 m/z) (Figure 8). For instance, the presence of the salicylic acid released from the (SR-*g*-PNVCL)-*g*-P2MBA films was confirmed by two methods.

**Figure 9:**
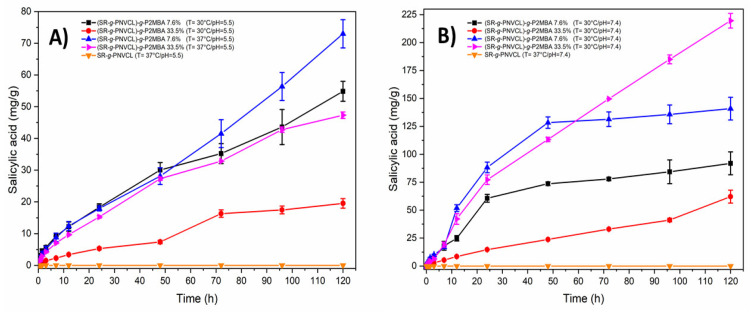
Salicylic acid release profile from (SR-*g*-PNVCL)-*g*-P2MBA films at (**A**) pH = 5.5 and (**B**) pH = 7.4.

Figure 9 shows the kinetics of salicylic acid hydrolysis from the modified (SR-*g*-PNVCL)-*g*-P2MBA films. The rate of hydrolysis was evaluated at 30 °C (below the UCST) and 37 °C (above the UCST), and pH 5.5 and 7.4 (physiological pH) [75]. In Figure 9A, films with a graft percentage of 7.6 and 33.5% at pH 5.5 show a sustained release for 120 h. Films with a 7.6% graft at 37 °C show 74.5 mg/g of drug released compared with 55 mg/g released at 30 °C (120 h). Faster hydrolysis at 37 °C (above the UCST) is due to the highest swelling of the films, favoring the interaction with the aqueous medium with the grafted polymeric chains of P2MBA. The films with 33.5% graft showed less drug release than the low grafts (7.6%), due to the lower swelling of the films. Additionally, grafting of 33.5% at 37 °C showed a faster release (48.3 mg/g) than at 30 °C (18.7 mg/g), at 120 h due to the differences in swelling at the temperatures studied. At pH 7.4 (Figure 9B), films with a graft of 7.6% at 30 °C presented a lower release (85.1 mg/g) than at 37 °C (135.8 mg/g) at 120 h, because of the differences in swelling at both temperatures. It is important to compare how these same films released a larger amount of the drug at pH 7.4 than at pH 5.5 since hydrolysis of polyester bonds (P2MBA) is favored by basic catalysis and the higher solubility of salicylic acid at higher pH [76]. It is observed that at 48 h, 7.6% graft releases more salicylic acid than 33.5% due to the higher swelling shown (Figure 7). However, at 72 h (pH 7.4), the process is reversed; films with 33.5% graft showed a release of 210 mg/g of salicylic acid at 37 °C, and 35.9 mg/g at 30 °C, at 120 h. This result is due to the higher content of prodrug in the higher grafted films.

It can be observed how a combination of films with high grafts, alkaline media (pH 7.4), and a temperature higher than 30.5 °C (UCST of the films) favors greater hydrolysis of salicylic acid grafted in the synthesized matrixes. With this, it was demonstrated that the synthesized materials are responsive to pH and temperature. Table 1 shows the kinetic models followed by the hydrolysis–release data. The results fit well to either zero-order or first-order kinetics. Table 1 shows the kinetic models followed by the hydrolysis–release data. The results fit well to either zero-order or first-order kinetics. Table 1 also presents the rate constants values. We observe a higher zero-order rate constant for the higher conditions (loading, pH, and temperature) studied. Three of the studies with lower loading follow first-order kinetics. Again, the higher constant value is observed for the higher pH and temperature studied.

### 3.4. Cytocompatibility Analysis 

**Figure 10:**
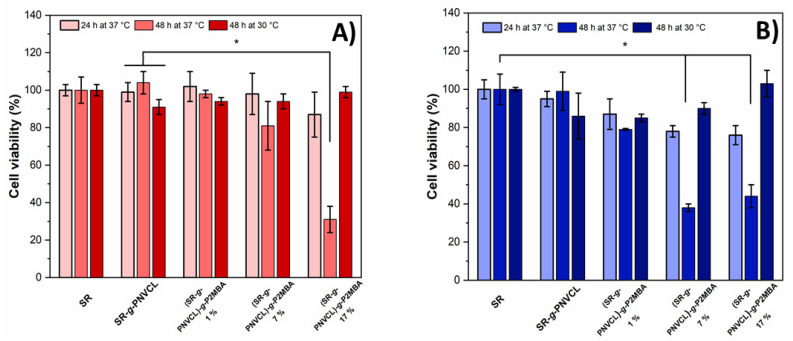
Viability of (**A**) BALB/3T3 and (**B**) HeLa cells in contact with SR, SR-*g*-PNVCL, and (SR-*g*-PNVCL)-*g*-P2MBA films. Asterisks indicate statistical significance between samples (*p* < 0.05).

Cell viability was assessed in small volumes of growing medium in direct contact with BALB/3T3 (mouse) and HeLa (human) cell lines in small volumes of growing medium (Figure 10). 

Cell viability was observed to be unaffected for very low-graft films (1%) in both cell lines, while good cell viability (>90%) was observed at 24 h for HeLa (Figure 10B) with 7 and 17% graft films, but not for BALB/3T3 cells (80%), presumably because a tumorigenic phenotypic cell such as HeLa can handle the microenvironmental acidity, a hallmark of cancer cells [77]. As previously reported, a high salicylic acid concentration decreases cell viability in a lymphocyte culture, in a pH-dependent way [78]. In the current study, a significant reduction in the cell viability (60%) was observed at 48 h for HeLa (Figure 10A) with 17% graft films, and for BALB/3T3 (Figure 10A) with 7 and 17% graft films, at 37 °C (Figure 10B). However, at 30 °C, no significant cell viability reduction was observed at any contact time for the 2MBA grafted materials. In this condition, the salicylic acid release is expected to be low because of the polymer’s reduced swelling. The results show that the polymer prodrug is responsive to temperature in a functional assay.

## 4. Conclusions

Modification of SR through two-stage graft polymerization was carried out, demonstrating that the concentration of a monomer (NVCL and 2MBA) and the radiation dose are critical factors to control the percentage of functionalization grafting. The characterizations by infrared spectroscopy and thermogravimetric analysis demonstrated the adequate incorporation of PNVCL in the first stage and P2MBA in the second stage. The scanning electron microscopy showed us how the graft was performed on the surface and bulk silicone. The swelling tests showed that the (SR-*g*-PNVCL)-*g*-P2MBA system had a UCST at 30.5 °C. Modified SR exposed to higher temperatures (above the UCST) demonstrated faster hydrolysis–release of salicylic acid, due to the higher swelling above the UCST, which favors contact of the material with aqueous media, triggering hydrolysis. These results are relevant since the faster release of the drug at higher temperatures proves the concept that materials with a UCST around body temperature can be used to control the release of anti-inflammatory drugs in response to fever caused by infections or in response to induced local heating [79]. The studies at different pH values showed that basic catalysis and higher solubility of salicylic acid at higher pH favors a more significant hydrolysis–drug release process. The cytocompatibility studies demonstrated how cancer and non-cancer cell viability is dependent on the environmental temperature. pH-/temperature-sensitive SR as a site-specific sustained-release polymeric prodrug system in medical devices is a promising strategy for prophylactic treatment in medical/surgical procedures.

## Figures and Tables

**Table 1:** Salicylic acid release kinetics data for (SR-*g*-PNVCL)-*g*-P2MBA films.

P2MBA Graft (%)	Release Medium pH	Temperature (°C)	Model	Rate Constants	R^2^
7.6	5.5	30	First order	0.015 h^−1^	0.991
33.5	5.5	30	Zero order	0.170 mg/g h	0.9696
7.6	5.5	37	Zero order	0.569 mg/g h	0.994
33.5	5.5	37	Zero order	0.401 mg/g h	0.978
7.6	7.4	30	First order	0.026 h^−1^	0.966
33.5	7.4	30	Zero order	0.469 mg/g h	0.985
7.6	7.4	37	First order	0.037 h^−1^	0.967
33.5	7.4	37	Zero order	1.867 mg/g h	0.9792

## Data Availability

Data sharing is not applicable to this article.
